# Studying Signaling Pathway Activation in TRAIL-Resistant Macrophage-Like Acute Myeloid Leukemia Cells

**DOI:** 10.32607/actanaturae.27317

**Published:** 2024

**Authors:** Y. V. Lomovskaya, K. S. Krasnov, M. I. Kobyakova, A. A. Kolotova, A. M. Ermakov, A. S. Senotov, I. S. Fadeeva, E. I. Fetisova, A. I. Lomovsky, A. I. Zvyagina, V. S. Akatov, R. S. Fadeev

**Affiliations:** Institute of Theoretical and Experimental Biophysics of the Russian Academy of Sciences, Pushchino, Moscow region, 142290 Russian Federation; Institute of Clinical and Experimental Lymphology, Branch of the Institute of Cytology and Genetics SB RAS, Novosibirsk, 630060 Russian Federation

**Keywords:** acute myeloid leukemia, TRAIL-induced apoptosis, transcriptome, inflammation

## Abstract

Acute myeloid leukemia (AML) is a malignant neoplasm characterized by extremely low curability and survival. The inflammatory microenvironment and maturation (differentiation) of AML cells induced by it contribute to the evasion of these cells from effectors of antitumor immunity. One of the key molecular effectors of immune surveillance, the cytokine TRAIL, is considered a promising platform for developing selective anticancer drugs. Previously, under *in vitro *conditions of the inflammatory microenvironment (a three-dimensional high-density culture of THP-1 AML cells), we demonstrated the emergence of differentiated macrophage-like THP-1ad clones resistant to TRAIL-induced death. In the present study, constitutive activation of proinflammatory signaling pathways, associated transcription factors, and increased expression of the anti-apoptotic *BIRC3 *gene were observed in TRAIL-resistant macrophage-like THP-1ad AML cells. For the first time, a bioinformatic analysis of the transcriptome revealed the main regulator, the *IL1B *gene, which triggers proinflammatory activation and induces resistance to TRAIL in THP–1ad macrophage-like cells.

## INTRODUCTION

Acute myeloid leukemia (AML) is a malignant blood disease characterized by extremely low curability and poor chance of survival [1]. Despite the progress made in therapeutic strategies over the past decade, the overall five-year survival rate is only 30% in patients diagnosed with AML [2]. AML is characterized by uncontrolled clonal expansion and accumulation (hypercellularity) of malignantly transformed hematopoietic progenitor cells in bone marrow and peripheral blood. It is well known that in acute myeloid leukemia, the bone marrow acquires the characteristics of damaged tissue, with signs of chronic inflammation [3, 4]. The inflammatory process in the bone marrow contributes to the avoidance of tumor cell death induced by both antitumor drugs and components of antitumor immunity; therefore, it is a marker of an unfavorable prognosis in the course of the disease [5, 6, 7]. It is also known that under inflammatory conditions, activation of proinflammatory intracellular signaling pathways can lead to myeloid differentiation of healthy hematopoietic progenitor cells [8, 9, 10]. Recently, there has appeared evidence that AML cells with a differentiated (mature) myeloid phenotype can suppress the activity of antitumor immunity and are more resistant to a number of antitumor drugs [11, 12, 13].

The apoptosis-inducing ligand (TRAIL), which is related to the tumor necrosis factor (TNF), is a key molecular component of antitumor immunity. Cytokine TRAIL binds to four membrane-bound receptors: pro-apoptotic DR4 and DR5, anti-apoptotic DcR1 and DcR2, and to the soluble anti-apoptotic “receptor” osteoprotegerin [14]. TRAIL is unique in its ability to selectively induce the death of tumors and transformed cells in the absence of cytotoxic effects on healthy cells. This property is very attractive and promising for the development of highly active agonists of pro-apoptotic TRAIL receptors, which, in turn, is extremely important for reducing any serious non-specific side effects of immunobiological antitumor drugs [15, 16].

Previously, we showed that in AML THP-1 cells under *in vitro *conditions, in a three-dimensional high-density culture simulating homotypic intercellular communication in the hyperplasia of leukemic blasts in the bone marrow, there was an increase in the production of proinflammatory cytokines, chemokines, and growth factors; activation of proinflammatory NF-kB-dependent signaling pathways; and a reversible increase in resistance to TRAIL-induced death and to the action of chemotherapeutic drugs [17, 18]. In addition, we have shown that macrophagelike clones THP-1ad with constitutive resistance to TRAIL-induced death appear in a three-dimensional high-density culture of these cells [19]. Differentiation of AML cells is also known to increase their resistance to TRAIL-induced death [20, 21].

Hence, based on the published data and our own results, we assume that the proinflammatory microenvironment of AML cells, simulated in a three-dimensional high-density cell culture *in vitro*, can induce cell maturation and lead to the emergence of new cell clones resistant to the cytotoxic effect of antitumor cytokine TRAIL. In this study, the bioinformatic analysis of the transcriptomes of macrophage-like THP-1ad cells that had formed under conditions of a proinflammatory microenvironment and were resistant to TRAIL-induced death identified the main signaling pathways and the key molecular participants associated with the activation of the survival pathways.

## EXPERIMENTAL

**Cell cultures**

The THP-1 human AML cell line (TB-202) was procured from ATCC (Manassas, VA, USA). Proliferating macrophage-like clones THP-1ad were obtained as previously described [19]. The cells were cultured in a RPMI 1640/F12 medium (Sigma, USA) supplemented with 10% fetal bovine serum (FBS) (Gibco, USA) and 40 µg/mL gentamicin sulfate (Sigma) at 37°C in the presence of 5% CO_2_. Non-proliferating macrophage-like THP-1PMA cells were obtained by incubating THP-1 cells with 200 nM phorbol-12- myristate-13-acetate (Sigma) for 96 h. For proinflammatory activation, THP-1 cells were cultured with 10 µg/mL LPS from *Escherichia coli *O111:B4 (Sigma) for 24 h.

**Cell transcriptome sequencing**

RNA sequencing for the analysis of cell transcriptomes was performed at Genoanalytika LLC using a HiSeq 1500 sequencer (Illumina, USA). RNA sequencing of each of the two groups of cells was performed in triplicate.

**Analysis of differential gene expression**

**Fig. 1:**
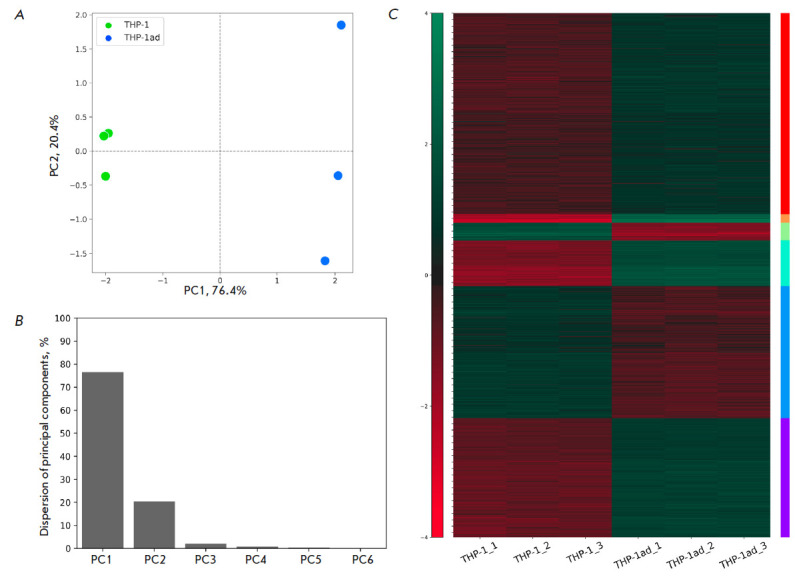
Comparative analysis of gene expression variations between THP-1ad and THP-1 cells. An analysis using principal component analysis was conducted for gene expression data to identify changes in expression between two experimental groups (*A*). Allocation of variation among the major components (*B*). Clustering of differentially expressed genes (DEGs) with significant changes in expression (*C*)

To identify differences in gene expression between macrophage-like clones THP-1ad and parent THP-1 cells, cluster analysis and principal component analysis using the Python programming language (v. 3.10.5) and the Scikit-learn package (v. 1.3.2) were performed (*Fig. 1*).

Gene set enrichment analysis (GSEA) was used to study the activation of signaling pathways in macrophage- like THP-1ad clones compared to parent THP-1 cells, since this method analyzes all the changing genes rather than only the genes with a multiplicity of changes above a certain threshold [22]. Gene sets from the H (Hallmark) and C3 collections (transcription factor target gene sets) of the MSigDB molecular signature database (https://www.gsea-msigdb. org/gsea/msigdb/human/collections.jsp) were analyzed using the Python programming language software package (v. 3.10) GSEApy (v. 1.0.5). The enrichment score was used as a criterion for the activation of signaling pathways and transcription factors (TF). Additionally, the normalized enrichment score (NES) was used to compare sets of genes containing different numbers of genes. The higher the value of the NES or enrichment score, the higher the probability of activation of the signaling pathway or TF is.

To identify differentially expressed genes (DEG), whose products can participate in the regulation of the studied signaling pathways, protein–protein interaction networks (PPIs) were constructed and their functional interactions were analyzed to identify the central regulatory elements. We used the STRING database (http://string-db. org), Cytoscape software (v. 3.10.0), and CytoHubba plugin [23].

**Quantitative reverse transcription PCR**

Total RNA was isolated using an innuPREP RNA Mini Kit 2.0 (Analytik Jena, Germany). cDNA was synthesized and amplified using the One Tube RT-PCR SYBR kit (Eurogen, Russia) on a QuantStudio 5 Real- Time PCR device (Thermo Scientific, USA), according to the manufacturer’s instructions. The oligonucleotide primers used in this study were synthesized at Eurogen CJSC and are listed in *Table 1*.

**Table 1:** The oligonucleotide primers used in this study

Oligonucleotide	The nucleotide sequence 5’→3’
NAIP-F	GGGGACTTCGTCTGGGATTC
NAIP-R	CTGGCCAGTGGAAGGAAAGT
CIAP1-F	CTGATTCCCGGCTCTGCG
CIAP1-R	AGCACGAGCAAGACTCCTTT
CIAP2-F	TCCATGGGTTCAACATGCCA
CIAP2-R	CTCCTGGGCTGTCTGATGTG
XIAP-F	TGGCGCTCATCGAGGGA
XIAP-R	TGTCTGCAGGTACACAAGTTTTAG
Survivin-F	TTCAAGGAGCTGGAAGGCTG
Survivin-R	GCAACCGGACGAATGCTTTT
BRUCE-F	AGAAAGGGATGATGCAAGTACG
BRUCE-R	CTACCTGGGCTGCTGAACTC
Livin-F	GGCCTCCTTCTATGACTGGC
Livin-R	GCAGAAGAAGCACCTCACCT
ILP-2-F	GGAGAGGAAAAGCGTTGTGC
ILP-2-R	TCTTCACTATGCATGGCGGG
BCL2-F	CAACATCGCCCTGTGGATGA
BCL2-R	CCGTACAGTTCCACAAAGGC
BCL2L1-F	GGCTTGTTCGGGAGAGACG
BCL2L1-R	CACTGAGTCTCGTCTCTGGTT
MCL1-F	TGGAGACCTTACGACGGGTT
MCL1-R	AGCACATTCCTGATGCCACC
BCL2L2-F	CGACTGTGACTCTGCTGCAA
BCL2L2-R	TCTCCCTGACTCGAGCTTTG
BCL2A1-F	GGATAAGGCAAAACGGAGGC
BCL2A1-R	TCTTCTTGTGGGCCACTGAC

**Statistical analysis**

Results are presented as a mean ± standard deviation (M ± SD). Experiments were performed with at least five repetitions (*n *≥ 5). The statistical significance of the differences was determined using one–sided ANOVA, followed by multiple Holm–Sidak comparisons (*p * < 0.05). The statistical significance of changes in gene expression was assessed using the Wald test adjusted for multiple Benyamini–Hochberg comparisons (FDR) ≤ 0.05 [24].

## RESULTS AND DISCUSSION

**Identification of the most activated signaling pathways in macrophage-like clones THP-1ad**

Previously, we demonstrated the formation of macrophage- like clones THP-1ad *in vitro *in three-dimensional high-density cultures of THP-1 AML cells with increased resistance to TRAIL-induced death [19]. To determine the main signaling pathways and potential mechanisms of TRAIL resistance in macrophage-like THP-1ad clones, the transcriptomes of these cells were sequenced, followed by an analysis of differential gene expression in comparison with the parent THP-1 cells.

To identify the activity of intracellular signaling pathways, gene set enrichment analysis (GSEA) of the MSigDB database [25] was performed on the entire transcriptome sequencing dataset, which allows one to analyze the activation/deactivation of the studied signaling pathway or all the target genes under study, because of the contribution of even minor changes in the transcriptional activity [22].

**Fig. 2:**
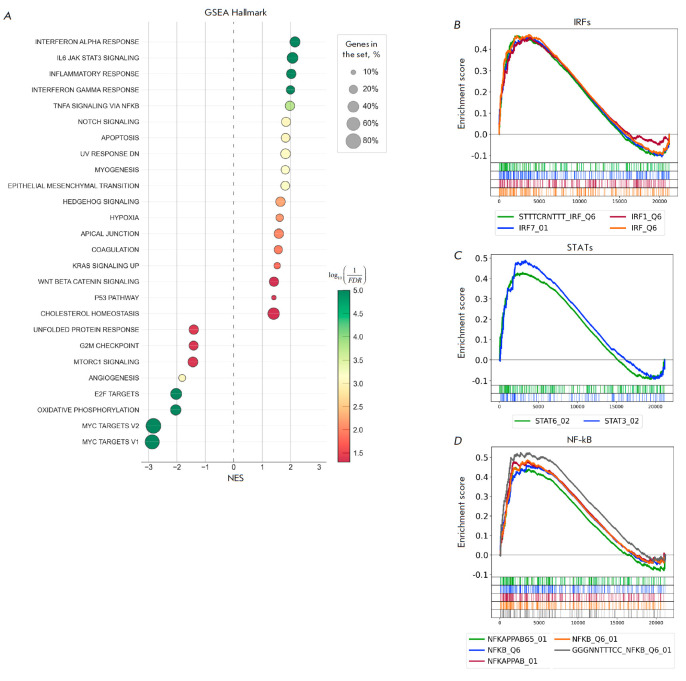
Comparative analysis of GSEA gene sets in THP-1ad macrophage- like clones and THP-1 parent cells. The GSEA findings are available for the H collection (*A*). The circle diameter is proportional to the number of genes exhibiting different expression levels compared to the total number of genes in the set. The GSEA yielded results for the C3 collection, specifically for gene sets (*B*, *C*, *D*). NES – normalized enrichment score. FDR ≤ 0.05

Gene set enrichment analysis using the H collection showed that in the macrophage-like clones THP-1ad, the sets of genes of the interferon alpha response (NES 2.13), IL6 JAK STAT3 signaling (NES 2.06), inflammatory response (NES 2.01), interferon gamma response (NES 1.98), and TNFA signaling via NF-KB (NES 1.96) had the highest positive normalized enrichment score (NES) (*Fig. 2A*), indicating activation of these signaling pathways. It is well known that the activity of the identified signaling pathways is characteristic of proinflammatory activation of cells [26, 27, 28]. We also found that in macrophage-like clones THP-1ad, the activity of the signaling pathways MYC targets V1 (NES -2.86), MYC targets V2 (NES -2.83), and oxidative phosphorylation (NES–2.03) was suppressed, as evidenced by the highest negative value of NES. Suppression of these signaling pathways is also characteristic of the proinflammatory activation of cells and increased resistance to TRAIL-induced death [29, 30, 31].

To identify the most probable TF controlling the expression of the genes from the sets with the highest positive NES value, that is, those directly involved in the activation of the aforementioned signaling pathways in macrophage-like clones THP-1ad, gene set enrichment analysis was performed using a C3 collection and sets of genes containing sequences for binding to TF of the IRF, STAT, and NF-kB families.

It was shown that of all the sets of the C3 collection (subcollections of TFT:TFT_LEGACY) containing genes binding TF of the IRF family, the sets of genes IRF_Q6 (ES 0.46), STTTCRNTTT_IRF_Q6 (ES 0.46), IRF1_Q6 (ES 0.45), and IRF7_01 (ES 0.45) were significantly (FDR ≤ 0.05) enriched and had a positive enrichment score (ES), which indicates the transcriptional activity of IRF1 and IRF7 factors. When studying sets containing genes with sequences for binding TF of the STAT family, it was found that the sets of STAT3_02 (ES 0.48) and STAT6_02 (ES 0.42) genes were significantly enriched (FDR ≤ 0.05) and had a positive ES, indicating the transcriptional activity of STAT3 and STAT6 factors. A study of the sets containing genes with sequences binding NF-kB showed that the sets GGGNNTTTCC_NFKB_ Q6_01, NFKB_Q6_01, NFKAPPAB_01, NFKB_Q6, and NFKAPPAB65_01 were significantly enriched (FDR ≤ 0.05) and had positive enrichment scores of 0.524001, 0.485919, 0.477002, 0.458895, and 0.44804, respectively, indicating an expressed NF-kB-dependent transcriptional activity (*Fig. 2 A–2D*).

The regulatory factors interferon IRF1 and IRF7 are known to regulate the expression of interferons of the first (α and β) and second (γ) types, acting as inducers of inflammation in the development of tumor diseases [32, 33, 34]. STAT3 and STAT6 are also known to be activated during inflammation in the tumor microenvironment; their activity may be associated with an increased inflammatory response during leukemia progression [35, 36, 37]. It has been posited that NF-kB plays a role in the formation of a leukemic microenvironment during the stimulation of a chronic inflammation in the BM niche under the effect of the cytokine tumor necrosis factor-α (TNFα), which supports a favorable environment for the survival and production of leukemic cells [38, 39, 40].

Hence, it can be assumed that proinflammatory signaling pathways associated with IFNα, IFNγ, IL-6, and TNFα are constitutively active in macrophage-like clones THP-1ad. In addition, the data obtained indicate that IRF1, IRF7, STAT3, STAT6, and NF-kB, the known modulators of tumor cell resistance, are involved in the activation of these processes.

**Identification of the potential regulatory genes involved in TRAIL resistance in macrophage-like THP-1ad clones**

**Fig. 3:**
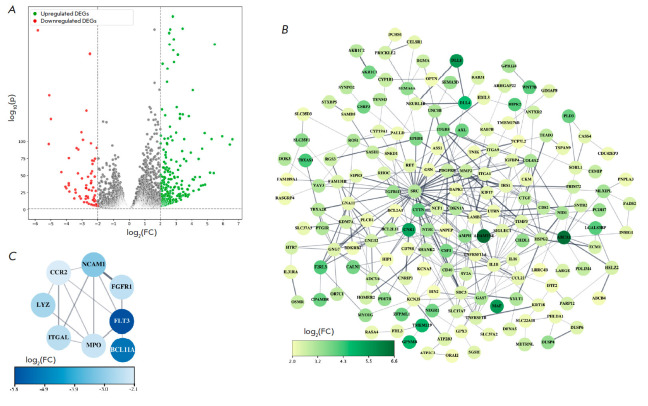
A diagram illustrating the distribution of DEGs in THP-1ad cells compared to the parent THP-1 cells (*A*). The PPI networks of DEGs products in THP-1ad cells are shown. The DEGs with increased expression are highlighted in green (*B*), whereas those with decreased expression are highlighted in blue (*C*)

To determine the DEGs whose products are most likely to act as regulatory elements of identifiable signaling pathways in macrophage-like clones THP-1ad from 21,511 transcribed genes, 355 DEGs were selected corresponding to the parameter 2 ≤ log2(FC) ≤ -2. Identification of the selected DEGs showed increased expression of 286 genes and decreased expression of 69 genes compared to those in the parent THP-1 cells (*Fig. 3A*).

PPI networks were built for DEGs with increased and decreased expression in order to identify interactions between DEGs products using the STRINGdb database [41]. It was shown that 167 out of the 286 genes with increased expression formed an interconnected network (*Fig. 3B*), whereas only eight out of the 69 genes with reduced expression formed a connected network (*Fig. 3C*).

**Fig. 4:**
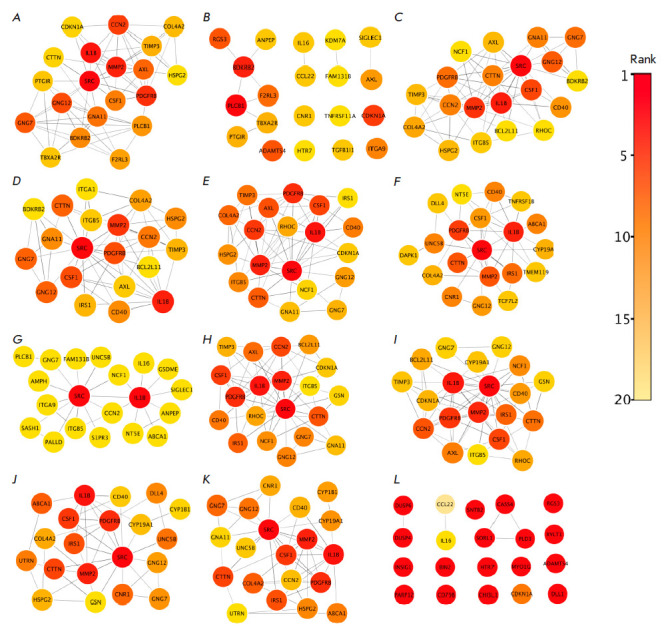
Clustering of the PPI network of genes with increased expression using the cytoHubba module algorithms: MCC (*A*), DMNC (*B*), MNC (*C*), Degree (*D*), EPC (*E*), BottleNeck (*F*), EcCentricity (*G*), Closeness (*H*), Radiality (*I*), Betweenness (*J*), Stress (*K*), and ClusteringCoefficient (*L*)

We analyzed the PPI network only for DEGs with increased expression, because it contained more interconnected participants than the DEGs network with reduced expression did, which could potentially make a more significant contribution to the formation of TRAIL resistance in macrophage-like THP-1ad clones. Clustering was then performed among the genes with increased expression in the PPI network using the cytoHubba module plug-in in the Cytoscape software [23]. Clustering was performed using 12 available cytoHubba algorithms to identify the most likely hub genes that could potentially contribute more to the formation of the PPI network and, thus, become potential targets for reducing TRAIL resistance in macrophage- like clones THP-1ad. Using cytoHubba algorithms, 20 genes with the highest rank value were identified in the analyzed PPI network. The rank in the PPI network shows the degree of “importance” of a gene, and the higher the rank (the closer to zero), the more significant this gene is for the formation of the network (*Fig. 4*).

In the PPI network clusters shown in *Fig. 4*, the five most repetitive genes were selected with the highest rank value, namely *CSF1*, which encodes a macrophage colonystimulating factor (M-CSF);* PDGFRB*, encoding the platelet growth factor receptor (PDGF); *MMP2*, encoding matrix metalloproteinase 2; *SRC*, encoding non-receptor tyrosine kinase SRC; and *IL1B *encoding interleukin1ß (IL-1ß). The role of the products of identifiable hub genes is well known in myeloid maturation, proinflammatory activation of cells, and progression of AML. M-CSF is the main regulator of macrophage differentiation and a promising target for AML therapy [42, 43]. The PDGF receptor has been shown to participate in the myeloid maturation of leukemic cells, activation of proto-oncogenic tyrosine kinases of the SRC family, and maintenance of the viability and proliferation of tumor cells [44, 45, 46]. SRC tyrosine kinases are the specific signaling integrators necessary for normal hematopoiesis and progression of acute leukemia [47, 48]. The role of IL-1β in the development of inflammatory processes and malignant neoplasms is well known. For example, chronic inflammation mediated by IL-1β is often associated with the emergence and progression of malignant tumors, as well as the direct regulation of myeloid cell differentiation and the signaling pathways that mediate the survival of leukemic cells [49, 50, 51]. Matrix metalloproteinases are involved in the migration of myeloid cells induced by an inflammation, and their suppression significantly reduces the viability and proliferation of AML cells [52, 53].

Therefore, the most probable regulatory genes of the signaling pathways activated in macrophagelike THP-1ad clones, such as *CSF1, PDGFRB, MMP2, SRC*, and *IL1B*, have been identified. The products of the identified genes can serve as promising targets for the suppression of TRAIL resistance in THP-1ad macrophage-like clones.

**Investigation of the interaction of hub genes with members of the IAPs and BCL-2 families**

It is well known that the main intracellular positive regulators of AML cell resistance to TRAIL-induced apoptosis are members of the BCL-2 and IAPs families, which block apoptosis at the mitochondrial and effector caspase levels [18, 54, 55]. PPI networks were built to determine the potential interaction of the identified hub genes with anti-apoptotic members of the IAPs and BCL-2 families.

**Fig. 5:**
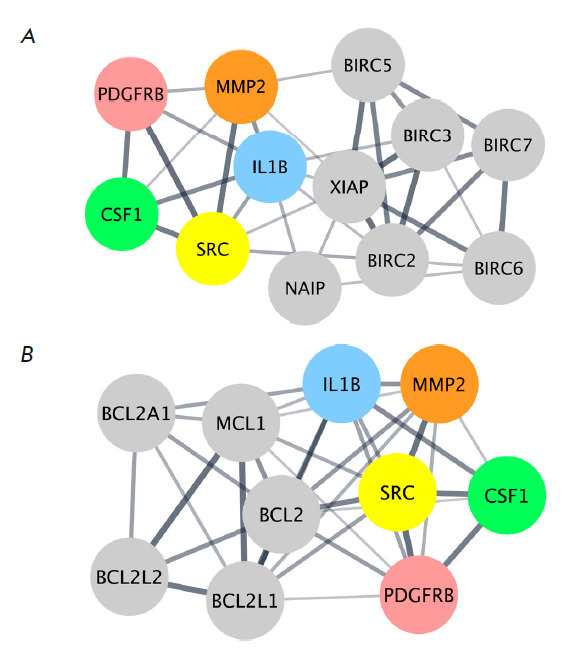
The discovered *IL1B, SRC, PDGFRB, MMP 2*, and* CSF1 *hub genes are connected in PPI networks with antiapoptotic members of the IAPs (*A*) and BCL-2 (*B*) families

Among the five identified hub genes, only *MMP2* (partners of *BIRC5 *and *XIAP*), *IL1B *(partners of* BIRC2, BIRC3, NAIP*, and *XIAP*), and *SRC *(partners of *BIRC2 *and *XIAP*) interacted with members of the IAPs family (*Fig. 5A*). Simultaneously, all the identified hub genes interacted with members of the BCL-2 family. The partners of the *IL1B *gene are* BCL2*, *BCL2A1*, and *MCL1*; the partners of the *SRC, PDGFRB*, and *MMP2 *genes are *BCL2, BCL2L1*, and* MCL1*; and that of the *CSF1 *gene is *BCL2* (*Fig. 5B*). Hence, all the identified concentrator genes can interact with anti-apoptotic members of the IAPs and BCL-2 families, which, in turn, indicates the potential participation of these families in the mechanism of resistance of macrophage-like THP-1ad cells to TRAILinduced death.

Furthermore, expression of all the anti-apoptotic members of the IAPs and BCL-2 family in macrophage- like THP-1ad clones and in parent THP-1 cells was revealed by quantitative reverse transcription PCR. Additionally, expression of these genes was analyzed in THP-1 cells treated with forbol ether (THP-1PMA) and LPS (THP-1LPS), known inducers of macrophage differentiation and activation of proinflammatory signaling pathways, respectively [56, 57].

**Fig. 6:**
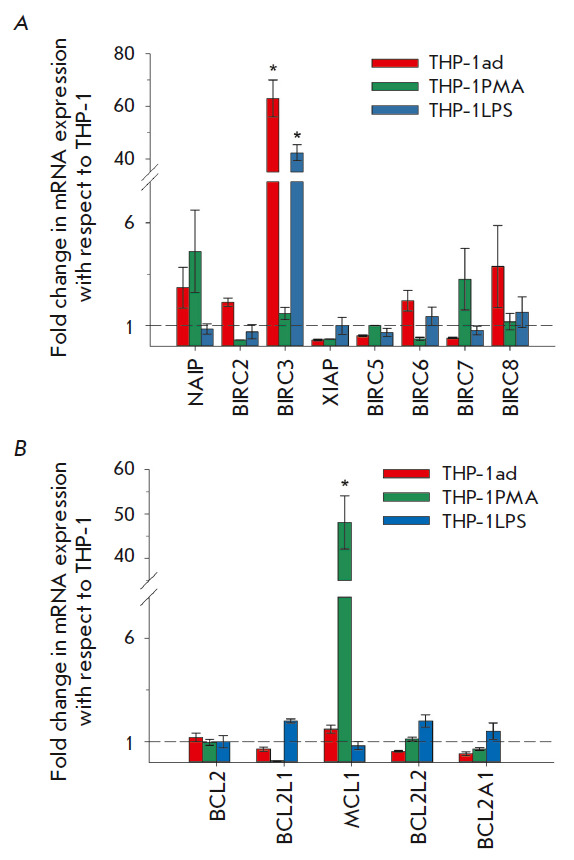
Gene expression of IAPs (*A*) and BCL-2 (*B*) family members was analyzed in macrophage-like THP-1ad clones, THP-1 PMA, and THP-1LPS cells. The data are presented as the mean value ± SD (*n *≥ 5). Statistical significance (*p ≤ 0.05) was observed when comparing the parent cells of THP-1

In THP-1ad cells, expression of only the *BIRC3* gene encoding the cIAP2 protein, an inhibitor of caspases 3, 7, 8, and 10, was significantly increased (63 ± 7 times) (*p *≤ 0.05) [58]. Similar results were obtained for THP-1LPS cells, and only the expression of the *BIRC3 *gene was also significantly (*p *≤ 0.05) increased (42 ± 3 times). No significant increase in the expression of IAPs family members was observed in THP-1PMA cells (*Fig. 6A*). An analysis of the expression of the BCL-2 family anti-apoptotic genes revealed a significant (*p *≤ 0.05) increase (48 ± 6 times) in the expression of the *MCL1 *gene. Inhibitors of the proapoptotic proteins Bax and Bak were expressed only in THP-1PMA cells (*Fig. 6B*) [59].

Therefore, in macrophage-like THP-1ad clones, the increase in the expression of the *BIRC3 *gene, which is a partner of the *IL1B *hub gene, is characteristic of proinflammatory activation, which most likely indicates the key role of this hub gene in increasing resistance to TRAIL-induced death.

The data on the activation of inflammatory processes in the bone marrow microenvironment in AML [5, 6, 60] and the role of cytokine IL1ß in the progression of myeloid leukemia have been reported [51, 61, 62]. In addition, macrophage differentiation is accompanied by increased expression of the cIAP2 protein [63]. However, these data on the possible participation of IL-1β-mediated proinflammatory activation in the development of the resistance of macrophage-like AML cells to cytotoxic TRAIL, potentially implemented through increased expression of *BIRC3*, were obtained here for the first time.

## CONCLUSION

Transcriptomic analysis of macrophage-like TRAIL-resistant THP-1ad clone AML cells, which were obtained under model conditions of the proinflammatory microenvironment of leukemic cells, showed high constitutive activity of the intracellular proinflammatory signaling pathways associated with IFNα, IFNβ, IL-6, and TNFα. The most probable TF, such as IRF1, IRF7, STAT3, STAT6, and NF-kB, have also been identified, potentially determining the activation of these signaling pathways. When searching for potential regulators of the identified proinflammatory signaling pathways, the most probable participants in these pathways, *CSF1, PDGFRB, MMP2, SRC*, and *IL1B*, were identified. It was also revealed that in THP-1ad cells, with proinflammatory activation, expression of the *BIRC3 *gene encoding cIAP2, an inhibitor of effector caspases, increased, which may mediate an increase in resistance to the cytotoxic TRAIL ligand. An important result is the discovery of a key molecular participant, the *IL1B *gene, which potentially links the processes of proinflammatory activation and the development of resistance to TRAIL in macrophage-like THP-1ad clones. Therefore, we believe that the mechanism of TRAIL resistance induction during the activation of inflammation in macrophage-like AML cells may consist of a IL-1β- associated, through NF-kB, increase in the expression of the inhibitor of apoptosis cIAP2.

## Abbreviations

AML: acute myeloid leukemia
TRAIL: tumor necrosis factor (TNF)-related apoptosis inducing ligand
TF: transcription factor
INF: interferon
IL: interleukin
FDR: false discovery rate
DEG: differentially expressed genes
PPI: protein-protein interactions
